# Expecting the unexpected: a review of learning under uncertainty across development

**DOI:** 10.3758/s13415-023-01098-0

**Published:** 2023-05-26

**Authors:** Selin Topel, Ili Ma, Jan Sleutels, Henk van Steenbergen, Ellen R. A. de Bruijn, Anna C. K. van Duijvenvoorde

**Affiliations:** 1 Leiden University, Institute of Psychology, Wassenaarseweg 52, 2333 AK Leiden, The Netherlands; 2 Leiden Institute for Brain and Cognition, Leiden, The Netherlands; 3 Leiden University, Institute for Philosophy, Leiden, The Netherlands

**Keywords:** Adolescence, Uncertainty, Volatility, Stochasticity, Learning, Decision-making

## Abstract

Many of our decisions take place under uncertainty. To successfully navigate the environment, individuals need to estimate the degree of uncertainty and adapt their behaviors accordingly by learning from experiences. However, uncertainty is a broad construct and distinct types of uncertainty may differentially influence our learning. We provide a semi-systematic review to illustrate cognitive and neurobiological processes involved in learning under two types of uncertainty: learning in environments with stochastic outcomes, and with volatile outcomes. We specifically reviewed studies (*N* = 26 studies) that included an adolescent population, because adolescence is a period in life characterized by heightened exploration and learning, as well as heightened uncertainty due to experiencing many new, often social, environments. Until now, reviews have not comprehensively compared learning under distinct types of uncertainties in this age range. Our main findings show that although the overall developmental patterns were mixed, most studies indicate that learning from stochastic outcomes, as indicated by increased accuracy in performance, improved with age. We also found that adolescents tended to have an advantage compared with adults and children when learning from volatile outcomes. We discuss potential mechanisms explaining these age-related differences and conclude by outlining future research directions.

**Supplementary Information:**

The online version contains supplementary material available at 10.3758/s13415-023-01098-0.

## Introduction

Uncertainty is a common feature of our everyday decisions and actions, and we must deal with incomplete information in many everyday situations. Despite its pervasiveness, uncertainty comes in many shapes and forms. For instance, think about trying a new coffee place; uncertainty may stem from not knowing some of the products offered, it may stem from uncertainty about the quality of their products, and it may even stem from uncertainty about the quality-stability of their products. At the counter, our decision may depend on what we expect to be the best at that time (i.e., oat milk cappuccino). Consequently, we may need to update those expectations or beliefs based on our experiences (i.e., How tasty was it?). Choosing the best course of action (i.e., Should I order this here again?) depends on our ability to learn from experiences and adjust our expectations accordingly by keeping track of outcomes and the changes in those outcomes over time. Specific periods in life, such as adolescence, have been characterized by being attuned to learning and navigating novel and inherently uncertain environments. This study is a review of recent literature on adolescent learning under different *types* of uncertainty.

Adolescence is a developmental phase between childhood and adulthood in which we transition into an adult role and develop mature social goals (Crockett & Crouter, 2014). The start of adolescence is biologically marked by the start of puberty, although the end of adolescence is less clearly defined (Sawyer et al., 2018). In Western societies, adolescence approximately spans the period between ages 10-24 years (including an age range sometimes referred to as emerging adulthood; Arnett, 2000; Sawyer et al., 2018; Jaworska & MacQueen, 2015). Puberty is characterized by a rapid rise in gonadal hormones, including testosterone and estradiol, which have a large influence on bodily characteristics, brain development, and behavior (Laube & van den Bos, 2016; Schulz & Sisk, 2016). Although the exact role of these hormones is unknown, conceptual models have hypothesized that pubertal hormones trigger the limbic brain system to flexibly recruit cortical control regions and potentially boost development of higher cognitive and self-regulatory functions important for learning. For instance, recent animal work has shown that hormonal levels directly influence the organization of the prefrontal cortex and accelerated performance in a reversal learning paradigm (Piekarski et al., 2017). Despite the link between hormonal changes and human learning under uncertainty still being inconclusive, it is suggested that these underlying neurobehavioral changes influence adolescent learning.

Additionally, adolescence is characterized as a life-phase in which individuals are confronted with new environments that may result in temporarily heightened uncertainty (Hartley & Somerville, 2015; Hofmans & van den Bos, 2022). For example, adolescents find themselves confronted with new social groups when beginning high school. They may also experience uncertainty about their social position within a new group and about newly formed social relationships that become more profound in adolescence. Adolescents may form deeper friendships, start romantic relations, or join certain groups outside of their family environment where they take on more roles and different responsibilities (Crockett & Crouter, 2014; Suleiman et al., 2017). The potential sensitivity to learning in the adolescent brain may help to rapidly reduce this heightened uncertainty and flexibly adapt behavior to new and changing environments (Crone & Dahl, 2012). A specific hypothesis that has been put forward is that adolescents may be more attuned to detecting changing outcomes over time and more readily adjust their behavior compared with children and adults (Lin & Wilbrecht, 2022; Romer et al., 2017). We examined this hypothesis by reviewing the developmental literature on learning under two types of outcome uncertainty: 1) stochastic outcomes, in which the outcome variation remains stable over time; and 2) volatile outcomes, in which there is a change in mean-value or probabilities of outcomes over time.

As highlighted in these examples, many changes in the adolescents’ environments may social, and it is debated whether learning from social and nonsocial outcomes relies on the same computational and neural mechanisms (Ruff & Fehr, 2014). Although studies revealed overlap in neural mechanisms when processing social and nonsocial rewards (i.e., a common currency; Corlett et al., 2022; Martinez-Saito & Gorina, 2022), there is evidence for a degree of specificity, in which parts of the prefrontal cortex (e.g., the dorsal medial prefrontal cortex, and lateral prefrontal cortex) may respond stronger or specifically to social than nonsocial learning outcomes (Corlett et al., 2022; Greimel et al., 2018; Martinez-Saito & Gorina, 2022; Apps & Sallet, 2017). However, given the limited set of studies in adolescents that contrasts learning under different types of uncertainty in social and nonsocial situations, we do not include this as a direct comparison in our review.

The goal of this review is to explore how the current available studies support the hypothesis that adolescence is particularly attuned to learning under uncertainty, in which we group studies based on their outcome stochasticity and outcome volatility. We first provide a definition of these different types of uncertainty and elaborate on our semi-structured literature search and inclusion strategy. Second, we discuss computational methods to understand the various manifestations of uncertainty, including the proposed neurobiological measures involved. Third, we review empirical evidence from age-related comparisons in studies examining learning under stochastic or volatile outcomes. Finally, we discuss the resulting implications for our understanding of adolescent learning under different types of uncertainty and present potential next steps for future research that also target individual differences.

## Different types of uncertainty: Stochasticity and volatility

**Fig. 1:**
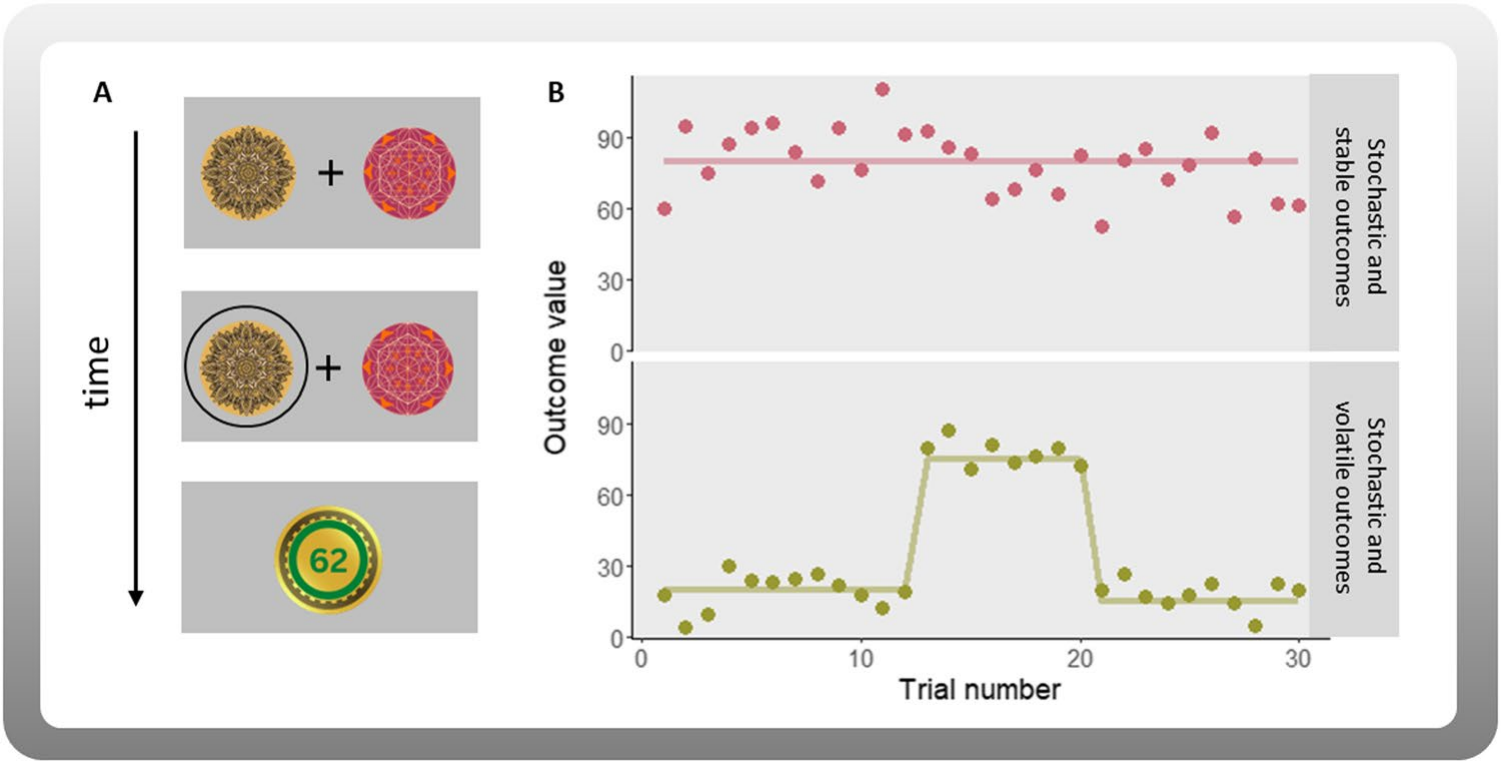
Example of a paradigm and outcome uncertainty types. **A** Stimuli-choice-outcome sequence. In a simple two-choice probabilistic learning paradigm (e.g., two-armed bandit task), participants are shown two options and asked to choose one. After making a choice, they see the outcome contingent on their action. Choosing the yellow fractal resulted in obtaining 62 points on this trial. The goal is to maximize reward by choosing the option that leads to better outcomes over the course of the task. This goal is achieved by learning from past outcomes. **B** Upper panel: the structure of a task environment where outcomes (e.g., number of points won) are distributed with some variance (SD = 15) around a mean value (M = 80), resulting in stochasticity. The mean value remains stable throughout the task. Due to the variance in the outcomes, this type of environment is characterized by high stochasticity. Similarly, the lower panel shows outcomes that are distributed around a mean value with variance (SD = 6), but the mean value (20 in the first 12 trials, 75 in the following 8 trials, 15 in the last 10 trials) changes throughout the experiment leading to increased volatility in addition to the stochasticity in the environment. To optimally adjust their learning speed, learners need to infer whether receiving an unexpected outcome (either a better- or worse-than-expected outcome) is caused by a change (due to volatility) or if it is a result of the random variance in the outcomes (i.e., due to stochasticity). We illustrated different types of uncertainty using continuous outcomes in this example, but other versions include similar setups with binary outcomes as well

The definition of uncertainty has sparked discussion in the literature. In general, uncertainty arises from outcome variability or incomplete information about the outcomes. Despite some conceptual overlap, different forms of uncertainty have been defined (Bland & Schaefer, 2012; Huettel et al., 2006; Piray & Daw, 2021; Pulcu & Browning, 2019; Soltani & Izquierdo, 2019; Yu & Dayan, 2005). Although other and more fine-grained distinctions have been made, we focus on *stochasticity*—also referred to as risk, expected or irreducible uncertainty—and *volatility*—sometimes referred to as unexpected uncertainty (e.g., differences between volatility and unexpected uncertainty; Bland & Schaefer, 2012). Both types of uncertainty can play a role in learning from repeated choices (e.g., learning task, Fig. 1A). To illustrate these different types of uncertainty in the lives of an adolescent, consider adolescents’ interactions. *Stochastic outcomes* refer to situations in which making the same decision may result in different outcomes (i.e., when there is outcome variance), a pattern that remains stable over time (Fig. 1B, upper panel). For example, meeting a friend after school is usually fun, but the friend is sometimes in a bad mood, which makes some interactions less enjoyable but still overall good. In contrast, *volatile outcomes* refer to situations in which the outcomes have changed, resulting in a new mean value and possibly different outcome variance (Fig. 1B, lower panel). For example, this friend has decided that they want to gain popularity in high school by joining a different social group and often is not friendly to you anymore. While seemingly dramatic, these examples are prevalent and representative of the lives of adolescents as this developmental phase comes with erratic mood changes (Maciejewski et al., 2019), formation of self-identity (Klimstra et al., 2010; Pfeifer & Berkman, 2018) and an increased importance of peer status and evaluation by peers (LaFontana & Cillessen, 2010; Sherman et al., 2016, 2018).

Making the distinction between stochastic and volatile outcomes is important. The literature on reinforcement learning suggests that these different types of uncertainty should optimally elicit different choice and learning strategies. That is, in an environment with high outcome stochasticity, an adaptive learner should integrate outcomes of their past decisions to form and update their internal value representation of the choice options. In an environment with high volatility, an adaptive learner should form and update expectations rapidly and based on recent outcomes after detecting a change (Behrens et al., 2007). Prior research has highlighted the importance for individuals to distinguish between volatility and stochasticity in their environment as these factors can interact (Piray & Daw, 2021; Yu & Dayan, 2005). For example, in environments with high estimated outcome stochasticity, unexpected events are more likely to be attributed to chance even when this event has occurred due to a real change. In other words, the ability to infer stochasticity and volatility can assist individuals in the challenging task of accurately estimating and responding to outcomes that arise from chance (stochasticity) versus those that indicate a change (volatility).

Despite the relevance of differentiating these two types of uncertainty, most developmental studies primarily focus on one of the two by making use of either probabilistic reinforcement learning paradigms targeting stochastic learning environments or reversal-learning paradigms targeting mainly volatility in learning environments, but also including a level of stochasticity. In this way, it could be suggested that these paradigms target, but do not isolate, learning under volatility. For a more detailed description of the task paradigms typically used in developmental and adult samples, see Box 1. Although relevant for understanding adolescent learning, the developmental literature is yet to distinguish between the behavioral and neural findings of learning under stochasticity and volatility. Therefore, as a first step, we reviewed and compared developmental studies that included an adolescent age range in stochastic learning contexts (without volatility) and volatile learning contexts (with or without stochasticity).

Box 1. Commonly used paradigms to study learning and decision-making under uncertainty

**Table:** 

**Probabilistic learning** Probabilistic learning paradigms commonly consist of two stimuli or actions to choose from, and depending on the underlying probabilities or contingencies, the choice leads to either a positive (e.g., reward, absence of punishment) or a negative outcome (e.g., punishment, absence of reward) with some variance. Thus, even after the associations are learned, it is not possible to always experience the same (rewarding) outcome due to the noise or stochasticity in this environment. For example, in such a learning paradigm, choosing one option could lead to a reward 80% of the time, whereas choosing the other leads to a reward only 20% of the time. These outcomes for the two options can be either perfectly anticorrelated or independent. **Reversal learning** Reversal learning paradigms are generally used to study cognitive flexibility and appear similar to the probabilistic learning paradigms. However, they require participants to detect when the contingencies for different options are reversed after every few trials (e.g., a previously more rewarding option becomes less rewarding and vice versa). There are versions of reversal paradigms with deterministic and probabilistic outcomes. In deterministic reversal learning paradigms, the better option leads to the reward 100% of the time when chosen and surprising outcomes signal a reversal. In probabilistic reversal learning paradigms, the surprising outcomes may indicate that the stimulus or action associated with reward most of the time has changed or it might be a result of stochasticity. The frequent contingency reversals increase the volatility in these task environments. Some of these paradigms introduce reversals only after certain criteria are met (e.g., choosing the more rewarding option at least three times in a row; Weiss et al., 2021). **Predictive inference** Instead of probabilities, a task environment might depend on more continuous outcomes, such as points gained or the location of a hidden stimulus. The outcomes of actions vary around a mean value, which leads to stochasticity. While estimating the underlying mean value, learners should not update their expectations too much due to these random fluctuations (e.g., estimation and choice tasks in Jepma et al., 2020). However, there also might be changes in the mean value that are not due to stochasticity in which case learners should update their predictions more quickly. In a task with continuous outcomes, volatility would be reflected as a the rate of change in the generative mean value (e.g., changepoint task; Nassar et al., 2016). **Risky decision-making** Finally, there may be alternative paradigms that include an element of *ambiguity* (unknown probabilities) or *risk* (known probabilities) when learning. These experience-based, decision-making tasks require participants to make choices between risky and safe(r) options that are presented (Christakou et al., 2013; Jepma et al., 2022; Nussenbaum et al., 2022; Rodriguez Buritica et al., 2019). Risky options are generally operationalized as the ones with greater outcome variability, and consistently choosing such risky options can be either beneficial or detrimental in the long run based on their average value (Jepma et al.,^ 2022^; Nussenbaum et al., 2022). Thus, both the average expected outcome values and variability (akin to stochasticity) should be learned or estimated over time for different options.	

## Neural and computational mechanisms of learning under uncertainty

Neuroscientists have described well-defined, reward-learning networks, including cortico-basal-ganglia loops, with the striatum and medial prefrontal cortex being key regions in this network (Haber & Knutson, 2010). The interpretation of learning signals in the brain has benefited from cognitive computational modeling that quantifies different parameters of learning that rely on deviations of expectations (i.e., prediction errors; Box 2). Rewards that exceed our expectations generate positive prediction errors, which can reinforce behavior. Conversely, worse-than-expected rewards generate negative prediction errors and lead to extinction of behavior. When the prediction error becomes zero, no further learning is possible and the prediction remains stable (Schultz, 2015). The extent to which a prediction error alters subsequent subjective valuation of choice options depends on one’s learning rate (Box 2). Prediction-error (PE) learning processes are assumed to depend on midbrain dopamine signaling (i.e., ventral tegmental area, substantia nigra) and their projections (Schultz, 2007; Schultz et al., 1997). Consistently, findings have pointed to a distributed network for prediction error coding, including dopamine-innervated regions, such as the striatum, ventral medial prefrontal cortex (PFC), and anterior cingulate cortex (Garrison et al., 2013), and also observed PEs in regions, such as the insula and lateral PFC (e.g., extensive meta-analysis on domain-general and domain-specific PEs, Corlett et al., 2022). It is debated whether there are specific brain networks involved in the updating of expectations (Bruckner et al., 2022), but at least one study has observed learning rates to be related to functional connectivity between the striatum and ventral medial PFC (van den Bos et al., 2012). Developmental research has aimed to quantify age-related changes in parameters of reinforcement-learning models and relate these to age-related changes in brain functioning. The use of computational models in different age groups in combination with brain measures may provide insights in how learning develops on multiple levels of explanation (Lockwood et al., 2020), but see recent reviews for discussion on the use of computational models in understanding learning processes (Nassar & Frank, 2016; Eckstein et al., 2021).

Another neurobiological framework on learning under uncertainty quantifies the importance of neurotransmitter systems, such as acetylcholine and noradrenaline (NA). Volatility, or unexpected changes, are thought to depend, at least partly, on the locus coeruleus-NA system (Bruckner et al., 2022). This system has been associated with uncertainty in Bayesian modeling approaches (Box 2) with rapid learning-rate adjustments. There is some evidence that inhibiting NA levels by using a pharmacological antagonist increases individuals’ learning rate through which beliefs about volatility are updated (Marshall et al., 2016). This indicates that NA stabilizes individual’s estimate of environmental volatility. Brain regions that have been related to coding uncertainty and surprise overlap partly with regions that are sensitive to prediction errors and include the anterior cingulate cortex (ACC; Behrens et al., 2007; d’Acremont & Bossaerts, 2016), posterior cingulate cortex (Payzan-LeNestour et al., 2013), and wider frontal-parietal brain regions (Kao et al., 2020). Other work has suggested the basolateral amygdala to be a key region for detecting outcome volatility, which may depend on the connections with the ACC, or potentially dopaminergic innervations (Soltani & Izquierdo, 2019). Overall, these findings point to a distributed network that includes regions of the PFC, parietal cortex, and subcortical regions involved in learning under uncertainty, as well as the neurochemical involvement of, at least, dopamine and NA. Many of these regions undergo large structural and functional development during adolescence and into adulthood (Silverman et al., 2015; Tamnes et al., 2017), and similarly changes in neurotransmitter systems are prevalent across adolescence (Larsen et al., 2020; Wahlstrom et al., 2010). It is at the moment, however, unclear how these changes contribute to adolescent learning under uncertainty.

Box 2. Computational models used to model learning and choice in their simplest form

**Table:** 

**Reinforcement learning (RL) models** In their simplest and widely used form, RL models include the Rescorla-Wagner learning rule (Equation 1b) combined with the Softmax choice function (Equation 2). An important element of learning in these models is the prediction error (PE), which is the difference between an expected (EV) and a received (O) outcome as a result of an action (e.g., choosing the right option) (Equation 1a). $PE_{t}=O{t}-EV(right)_{t}\qquad\qquad\qquad(1 \text a)$ $EV(right)_{t+1}=EV(right)_{t}+a(PE_{t})\quad(1 \text b)$ Parameters in RL models that are estimated from the data and used to calculate PEs are a learning rate (*α*) and decision temperature (*β*). Learning rates reflect the degree of updating of expectations (EV), i.e., expected value of a stimulus or action. The EV is then updated for each stimulus or action separately at time *t* (note that there might be variations of these models where EVs for both options are updated simultaneously based on the outcome received for one of them, i.e., when the options are perfectly anticorrelated). Although these models can be extended in several ways, one common version includes separate learning rates for positive (better-than-expected) and negative (worse-than-expected) PEs. $p{(right)}_t=\frac{1}{1+{e}^{-\beta \left( EV{(right)}_t- EV{(left)}_t\right)}}\kern2.25em (2)$ The choice is then determined by the Softmax function, which assigns higher choice probability to the option with the higher EV proportional to the difference of the EVs for different options with varying sensitivity (Equation 2). The decision temperature (also called inverse temperature) indicates the degree of this sensitivity and can indicate more or less exploratory choice behavior depending on its value. Learning rates determine how much influence PEs have on the updating; a higher learning rate would lead to larger influence of the most recent outcomes, whereas a lower learning rate would lead to slower integration across a history of multiple outcomes. **Bayesian Updating Models** Simple reinforcement-learning models do not incorporate uncertainty directly in their computational framework. In contrast, Bayesian models assume that individuals attempt to infer the environment’s hidden states given an individual’s observations (i.e., given the outcomes). In Bayesian models, uncertainty is explicitly built in. That is, in Bayesian learning models, there is not a single estimation of EV, but there is a belief distribution over the world state of interest given the observations. This belief distribution starts with a prior belief distribution and is updated with each observation based on Bayes rule, resulting in the posterior belief distribution of an individual. The posterior distribution is then used in the decision rule by maximizing the expected utility under the posterior (e.g., maximum a posteriori (MAP) decision rule), while the width of the distribution corresponds to uncertainty about the environment’s state. For more information see e.g. Ma et al. (2022a).	

## Semi-systematic review approach

Semi-systematic literature reviews are used to integrate evidence on topics that are conceptualized and studied in different ways which may impede the process of a full systematic review and/or meta-analysis (Snyder, 2019). We opted for a semi-systematic review to study the developmental differences in learning performance and strategies from stochastic and volatile outcomes by making use of an extant literature including a diverse set of studies on belief updating and reinforcement learning. Thus, we searched terms on the PubMed database related to *Uncertainty, Probability Learning, Reversal Learning, Reinforcement*; together with terms, such as *Developmental, Adolescent Development, Young Adult, Puberty* (final search date July 26, 2022; see full list of terms in Supplementary Tables S1-2). In addition to screening these articles published from 2010 (excluding review articles, studies that did not include adolescent samples and those that did not include any age-related analyses), we also used snowballing methods by searching for the citing papers of these articles, and articles cited by them to identify other relevant papers and preprints (Supplementary Figure S1, flow diagram). Tables 1 and 2 summarize all studies, including age ranges and paradigms, model parameters, and whether neuroimaging data were included. Supplementary Table 1 includes the means of parameters estimates in the studies (if reported). We discuss the studies of learning under stochasticity (Table 1) and volatility (Table 2) separately in relation to age-related differences in behavioral, computational modeling, and neural findings and make suggestions for future studies.

**Table 1:** Studies that investigated developmental differences in learning and decision-making with stochastic outcomes

Author (yr)	Task type	Task details	Age group (N)	Parameters*	Performance / accuracy	fMRI
Christakou et al. (2013)	Adapted Iowa Gambling Task (IGT)	Stimuli were four decks of cards: Decks A and B gave relatively large gains (£190, £200, or £210) but even larger losses (£240, £250, or £260), whereas Decks C and D made small gains (£90, £100, or £110) but even smaller losses (£40, £50, or £60). There was a 50% probability of winning or losing on each deck. Consequently, Decks A and B were disadvantageous (also referred to as “risky”), because they led to a net loss on average. Decks C and D were advantageous (also referred to as “safe”), because they led to a net gain.	11.9–31.2 (N_adolescents_ = 18; N_adults_ = 19)	Positive learning rate: Decreased with age Negative learning rate: Increased with age (only in adolescent not adult group) Inverse temperature: adults > adolescents	Task performance improved significantly during adolescence, stabilizing in adulthood.	Yes
Cohen et al. (2010)	Probabilistic learning	Stimuli consisted of two types of abstract stimuli (predictable and random) that needed to be categorized into one of the two categories (Northern and Eastern). If they were categorized, the predictable and random stimuli led to reward 83% and 50% of the time, respectively.	Children: 8-12 (*N* = 18) Adolescents: 14-19 (*N* = 16) Adults: 25-30 (*N* = 11)	Learning rates: No difference Inverse temperature: NA	Increased with age.	Yes
Davidow et al. (2016)	Probabilistic learning	The Butterfly task combined with a memory task testing the ability to recall images shown at the time of feedback receipt. One of four cues, and two targets presented on each trial. Each cue was associated with one target 80% of trials and with the other 20% of the trials.	Adolescents: 13-17 (*N* = 41; N_fMRI_ = 25) Adults: 20-30 (*N* = 31; N_fMRI_ = 22)	Learning rate: adults > adolescents Inverse temperature: No difference	Adolescents performed better than adults.	Yes
Decker et al. (2015)	Instructed probabilistic learning	Participants saw one of three stimulus pairs on each trial, (AB, CD, and EF; adapted from Frank et al., 2004). Positive and negative FB: AB pair (80:20%), CD pair (70:30%), EF (70:30%), but they were given incorrect instruction about the F.	Children: 6-12 (*N* = 30) Adolescents: 13-17 (*N* = 31) Adults: 18-34 (*N* = 26)	Learning rate: children > adolescents and adults Inverse temperature: increased with age	Children performed worse than adults, and trend-level worse than adolescents. Adolescents’ and adults’ performance did not differ significantly.	No
Hämmerer et al. (2011)	Probabilistic learning	One of three stimulus pairs on each trial, (AB, CD, and EF; adapted Frank et al., 2004) was presented. Outcomes were either gaining or losing 10 points. Three types of choice pairs with different reward probabilities: 85:15%; 75:25%; 65:35%.	Children: 9-11 (*N* = 44); Adolescents: 13-14 (*N* = 45) Younger adults: 20-30 (*N* = 46) Older adults: 65-75 (*N* = 44)	NA	Relative to adolescents and young adults, both children and older adults performed worse.	No
Humphreys et al. (2016)	Balloon Emotional Learning Task (BELT) (Modified Balloon Analogue Risk Task [BART])	Three different conditions with different balloon explosion points were used: slow-to-explode (19 pumps), variable (7, 13, or 19 pumps), and quick-to-explode (7 pumps) balloons. Participants pumped up balloons to earn points for 27 balloon trials. Participants could learn over time to determine how much they should inflate the balloons while avoiding explosions.	Children and adolescents: 3-17 (*N* = 140) Adults: 18-36 (*N* = 76)	NA	Performance (points gained and learning over time) increased with age.	No
Jepma et al. (2020)	Estimation and choice tasks	In the estimation task, participants estimated the average number of points in a box (i.e., generative mean), rated their certainty (1-10 scale), and were shown a number drawn from the box. The SD was either 4 or 8 leading to some noise in observations. In the choice task, participants chose between two stimuli. Chosen stimulus led to some points and the amount of points for each stimulus determined by a Gaussian distribution with a fixed mean, SD was 8.	Adolescents: 12-15 (*N* = 25) Adults: 18-29 (*N* = 35)	Learning rate: (dynamic) asymptote adolescents > adults Inverse temperature: adults > adolescents	Higher performance in adults vs. adolescents.	No
Jepma et al. (2022)	Experience-based risk taking	Task required learning in risk advantageous vs. risk disadvantageous context. Two choices (risky vs. sure) were presented. Sure one always gives the same small reward; risky one gives either large reward or no reward. The only variable that needed to be learned was the reward probability of the risky option. Three conditions (10 blocks 4 risky-disadvantageous, 4 risky-advantageous, 2 risky-neutral): EV of the risky option is lower, higher or equal to the sure option. Each block has 20 trials with outcomes +10, 0, +20. Sure vase contained balls with +10 label, risky vase had balls with 0 and +20 on them.	Early adolescents: 12-14 (*N* = 31) Mid-late adolescents: 15-17 (*N* = 39) Adults: 20-35 (*N* = 35)	Learning rate: early adolescents > late adolescents; Adults had a dynamically decreasing LR started similar to late adolescents, but decreased more, over trials Inverse temperature: adults > adolescents, no difference between late and early adolescents.	Adults optimized their risky choice behavior faster than mid-late adolescents who in turn optimized faster than early adolescents.	No
Jones et al. (2014)	Social evaluative reinforcement learning	One of three stimuli (fictitious peers) with different probabilities of social reward (33%, 66%, 100%) was presented. Outcome: received social reward, or someone else received the reward (absence of reward). RTs used as the index for learning.	8-25 (*N* = 120; N_fMRI_ = 87)	Positive learning rate: children & adults > adolescents, decreased with age Negative learning rate: No difference Inverse temperature: NA	Accuracy increased with age.	Yes
Nussenbaum et al. (2022)	Adapted IGT	Similar to Christakou et al. (2013), four decks of cards with two risky and two safer decks, but with two contexts: one where risky options had higher average value (25), another where they had lower average value (−25). In each block, 100 trials were completed. Probability of gain and loss was 50%. Goal was to earn as many points as possible.	Children: 8-12 (*N* = 47) Adolescents: 13-17 (*N* = 46) Adults: 18-25 (*N* = 49)	Positive learning rate: No difference Negative learning rate: Decreased with age Inverse temperature: increased with age	Older participants performed better.	No
Palminteri et al. (2016)	Instrumental probabilistic RL	Two stimuli were presented. Both outcome valence (Reward vs. Punishment) and feedback type (Partial vs. Complete) were manipulated using a within-subjects factorial design. Probabilities were 75% and 25%. Gain led to 1 point; loss led to losing 1 point.	Adolescents: 12-17 (*N* = 26) Adults: 18-32 (*N* = 24)	Learning rate: No difference Inverse temperature: adults > adolescents	Adolescents displayed reduced punishment learning performance compared to adults.	No
Raab and Hartley (2020)	Probabilistic Go/No-Go learning	Participants decided whether to press the button via a keyboard press (“Go” response) or not press the button (“No-Go” response). Outcomes were probabilistic: 80:20% reward.	Children: 8–12 (*N* = 20) Adolescents: 13–17 (*N* = 20) Adults: 18–25 (*N* = 21)	Learning rate: No difference Reinforcement sensitivity: Increased with age Inverse temperature: NA	Peak in overall task performance during late adolescence (16-17).	No
Rodriguez Buritica et al. (2019)	Adapted IGT	Stimuli were four decks of cards: Two decks were good decks (higher expected value) and two were bad decks (lower expected value). They begin by receiving advice from a same-aged peer for a good deck.	Children: 8-10 (*N* = 24) Adolescents: 13-15 (*N* = 24) Adults: 18-22 (*N* = 25)	Positive learning rate: No difference Negative learning rate: children > adolescents & adults Inverse temperature: Increased with age	Adolescents and adults performed better than children.	No
Rosenblau et al. (2018)	Social learning/ Theory of Mind (ToM)	The task required learning about other people’s mental states (their preferences for activities, fashion, and food items). Following their rating (likes: 1 to 10) for an item, they received trial-by-trial feedback about the other’s actual preference rating.	Adolescents: 10-17 (*N* = 24) Adults: 23-36 (*N* = 21)	Learning rate: early adolescents and adults > mid adolescents (13-15) Inverse temperature: NA	Adults vs. adolescents made more accurate predictions.	Yes
Smith et al. (2012)	Computerized IGT	Four decks of cards (A, B, C, and D) are presented to choose from. Two decks lead to large gains but even larger losses (disadvantageous; A and B), and the other two are associated with smaller gains but even smaller losses (advantageous; C and D). Performance is indicated by net scores (subtracting choices for disadvantageous decks from choices for advantageous decks).	8-17 (*N* = 122)	NA	Children and mid-late adolescents performed better than early adolescents (ages 10-13).	No
van den Bos et al. (2012)	Probabilistic learning	One of two stimulus pairs (AB and CD), everyday objects were presented. Choosing A in AB leads to positive feedback on 80%, choosing B leads to positive FB 20% of the trials. For CD, these were 70% and 30%.	Children: 8-11 (*N* = 18) Adolescents: 13-16 (*N* = 27) Adults: 18-22 (*N* = 22)	Positive learning rate: Marginally increased with age Negative learning rate: Decreased with age Inverse temperature: No difference	Not reported	Yes
Westhoff et al. (2020)	Repeated probabilistic trust learning	Task consisted of repeated one-shot interactions with members of two groups (trustworthy vs. untrustworthy). Outcomes were 73% cooperative in the trustworthy environment.	8-23 (*N* = 244)	Learning rate: 8-11-year-olds had a stable LR (higher on average); In the older age cohorts, learning rates start high and decay over trials Inverse temperature: Not reported	Not reported	No
Westhoff et al. (2021)	Prosocial learning	Stimulus pairs were presented for each of three conditions: the monetary consequences impacted self, other, and no one. One stimulus was associated with winning 1 point with 75% probability and another with 25% probability.	9-21 (*N* = 74)	Learning rate: Decreased with age Inverse temperature: Increased with age	Performance improved with age, particularly when learning for others.	Yes
Xia et al. (2021)	Probabilistic learning	The Butterfly task (Davidow et al., 2016) with 80% and 20% probability of preference for a flower per butterfly. Correct feedback led to 1 point; incorrect feedback led to 0 point.	Children and adolescents: 8-17 (*N* = 157) Adults: 18-30 (*N* = 118)	Positive learning rate: Increased with age Inverse temperature: Increased with age	Performance increased with age through early 20s and then stabilized.	No

*Where results regarding parameters are entered as nonapplicable (NA), either the studies did not employ computational models or the fitted models did not include these parameters. If the analyses related to a given parameter were not reported in the papers, then the parameter values were entered as “not reported.” Mean (or median) values of parameters are reported in Supplementary Table S3

**Table 2:** Table outlining studies that investigated developmental differences in learning and decision-making with volatile outcomes

Author (yr)	Task type	Task details	Age group (N)	Parameters*	Performance / accuracy	fMRI
Bruckner et al. (2020)	Predictive inference	Helicopter task (Nassar et al., 2016) with variability and change points in two versions.	Children: 8-10 (*N* = 33) Adolescents: 12-17 (*N* = 29) Young adults: 20-28 (*N* = 32) Older adults: 62-80 (*N* = 35) 2^nd^ study: Children: 7-11 (*N* = 31) Young adults: 20-28 (*N* = 25) Older adults: 61-76 (*N* = 34)	Learning rate: children & older adults < adolescents and younger adults Inverse temperature: NA	Children and older adults vs. adolescents and adults made more errors. Children and older adults did not differ; adolescents and younger adults did not differ.	No
Eckstein et al. (2022)	Probabilistic (reward) reversal learning	Choice between one of two boxes that contained the coin. The box where the coin appeared switched unpredictably: volatility. Correct location did not always provide coins: stochasticity. Correct location was rewarded 75%; incorrect location was never rewarded. Outcome was either gain or no gain. 120 trials, 5-9 switches.	Children and adolescents: 8-18 (*N* = 179) Adults: 18-30 (*N* = 112)	Positive learning rate: largest in adults Negative learning rate: smallest in adolescents Inverse temperature: Increased monotonically with age	Peak performance for 13-15 y/o.	No
Hauser et al. (2015)	Probabilistic reversal learning	Two stimuli were presented. Reversals occurred after a minimum of 6 correct responses – 3 consecutive correct responses. Correct choices rewarded 80% of the time; incorrect rewarded 20%. Outcomes were either loss or gain.	Adolescents: 12-16 (*N* = 19) Adults: 20-19 (*N* =17)	Positive learning rate: No significant difference Negative learning rate: adolescents > adults Inverse temperature: No difference	No performance differences.	Yes
Javadi et al. (2014)	Probabilistic reversal learning	Two stimuli were presented. Probabilities reversed after at least 4 consecutive correct responses with a probability of 0.25. 120 trials, two stimuli. Correct stimulus led to a reward (+20 cents) 70% of the time, and a loss (−20 cents) 30% of the time. The wrong stimulus led to a reward (+20 cents) 40% of the time and loss (−20 cents) 60% of the time.	Adolescents: 14-15 (*N* = 219) Adults: 20-39 (*N* = 29)	Learning rate: No difference; Inverse temperature: adults > adolescents	No performance differences.	Yes
van der Schaaf et al. (2011)	Deterministic reversal learning	Two stimuli, a face and a scene, were presented vertically, each associated with either reward or punishment. One stimulus was highlighted, the participants reported whether this would be associated with reward or punishment. Reward was a green smiley, “+100 euro” sign and a high-frequency jingle tone; punishment was a red sad smiley, “−100 euro” sign, and a low-frequency tone. Outcomes did not depend on participants’ responses. Reversals occurred after 4, 5, or 6 correct responses.	Children: 10-11 (*N* = 13) Younger adolescents: 13-14 (*N* = 14) Older adolescents: 16-17 (*N* = 15) Adults: 20-25 (*N* = 16)	NA	Overall, children performed worse vs. older age groups, no difference between adolescents and adults. In reversal trials, peak performance was in adolescence (13–17 y/o).	No
Waltmann et al. (2023)	Probabilistic reversal learning	Two contexts: winning rewards, avoiding loss. Binary choices between two cards: the cards are associated with different probabilities of winning (+10 cents) or not winning (±0 cents) (80%-20%) in the win block and of losing (−10 cents) or not losing (±0 cents) in the loss block. Five reversals after the initial acquisition phase.	Adolescents: 12-18 (*N* = 40) Adults: 19-45 (*N* = 55)	Learning rate: no difference Positive reinforcement sensitivity: adults > adolescents Negative reinforcement sensitivity: no difference Inverse temperature: NA	No difference overall; adolescents tended to perform worse than adults on a few trials before reversal and better on a few trials after reversal.	Yes
Weiss et al. (2021)	Probabilistic reversal learning	Two stimuli were shown. Received positive feedback after correct choice with 75% and negative feedback with 25% probability. After reaching a learning criterion, these reversed. Learning criterion was to complete at least 6-10 trials and respond correctly on three consecutive trials.	Children: 8-12 (*N* = 28) Adolescents: 13-17 (*N* = 25)	Learning rate: adolescents > children; Inverse temperature: No difference	Children vs. adolescents made more errors.	No

*Where results regarding parameters are entered as nonapplicable (NA), either the studies did not employ computational models or the fitted models did not include these parameters. Where the analyses related to a given parameter were not reported in the papers, the parameter values were entered as “not reported” in the table. Mean (or median) values of parameters are reported in Supplementary Table S3

## Results

### Development of learning from stochastic outcomes

Table 1 lists empirical studies comparing developmental samples using tasks that involve outcome stochasticity. The majority of these studies employed probabilistic learning tasks with stable reward contingencies, and a few used experience-based decision-making tasks. Among these are studies that used a RL model (except Hämmerer et al., 2011; Humphreys et al., 2016; and Smith et al., 2012) and studies with (*n* = 7) or without (*n* = 12) neuroimaging. Only four of the reviewed studies with stochastic but stable outcome contingencies used social rewards or feedback, and the nature of these were highly diverse (i.e., prosocial reward, reciprocity of trust, acceptance, and feedback about others’ mental states), hindering our ability to directly compare social to nonsocial tasks.

When summarizing these developmental findings, we first consider how quickly individuals at different ages update values of different stimuli or actions in contexts with stochastic but otherwise stable outcomes which should evoke higher degrees of expected uncertainty. These findings have been mixed. Whereas some studies reported that adolescents had lower learning rates than adults (Davidow et al., 2016; Jones et al., 2014; Rosenblau et al., 2018; Xia et al., 2021), others reported a decrease in learning rates with age (Decker et al., 2015; Jepma et al., 2020; van den Bos et al., 2012; Westhoff et al., 2020, 2021) or no age-related differences (Palminteri et al., 2016; Raab & Hartley, 2020). A subset of these studies (*N* = 6) reported asymmetrical learning rates for positive (better-than-expected) and negative (worse-than-expected) PEs—referred to as positive and negative learning rates in short—instead of single learning rates, which further added to divergent findings in the literature (Christakou et al., 2013; Jones et al., 2014; Nussenbaum et al., 2022; Rodriguez Buritica et al., 2019; van den Bos et al., 2012; Xia et al., 2021). If we look at these separately, however, positive learning rates in children and adolescents showed mixed findings. One study reported higher positive learning rates in children and adults relative to adolescents (Jones et al., 2014); another reported a marginal increase with age from childhood to adulthood (van den Bos et al., 2012). Two studies reported opposite patterns: one reported a decrease in positive learning rates from early adolescence to adulthood (Christakou et al., 2013), and the other reported an increase (Xia et al., 2021). Yet others reported no difference in positive learning rates across ages (Nussenbaum et al., 2022; Rodriguez Buritica et al., 2019). Negative learning rates seemed to be relatively more consistent where children showed either the highest (Nussenbaum et al., 2022; Rodriguez Buritica et al., 2019; van den Bos et al., 2012) or similar levels (Jones et al., 2014) compared with other age groups. Adolescents showed similar negative learning rates to adults (Jones et al., 2014; Rodriguez Buritica et al., 2019) or negative learning rates decreased with age (Nussenbaum et al., 2022; van den Bos et al., 2012), except for one study that reported an increase in negative learning rates with age in adolescence but not in adulthood (Christakou et al., 2013).

In contrast, findings from most studies indicate a decrease in choice stochasticity and exploration (i.e., inverse temperature) in adults compared with children and adolescents in most studies (Decker et al., 2015; Jepma et al., 2020; Nussenbaum et al., 2022; Palminteri et al., 2016; Rodriguez Buritica et al., 2019; Westhoff et al., 2021; Xia et al., 2021; but see Davidow et al., 2016, and van den Bos et al., 2012). Moreover, learning performance—as indicated by the proportion of choices for the option with higher underlying mean value, correct responses, or more accurate predictions depending on the task characteristics—generally increased with age (Christakou et al., 2013; Cohen et al., 2010; Humphreys et al., 2016; Jepma et al., 2020; Jepma et al., 2022; Jones et al., 2014; Nussenbaum et al., 2022; Palminteri et al., 2016; Rosenblau et al., 2018; Westhoff et al., 2021; Xia et al., 2021) . Compared with the number of studies that reported an age-related increase in learning performance, fewer studies reported a decrease in performance from adolescence through adulthood (Davidow et al., 2016; Raab & Hartley, 2020), or they reported no age-related differences between adolescents and young adults (Rodriguez Buritica et al., 2019) but found that children and older adults performed worse than adolescents and young adults (Decker et al., 2015; Hämmerer et al., 2011). One study also found a U-shaped relationship with age from childhood to mid-late adolescence with lowest performance between ages 10-13 years (Smith et al., 2012).

Neuroimaging findings show that in a learning context with outcome stochasticity, PEs scale with the activity in the ventral striatum, and medial PFC (Cohen et al., 2010; Davidow et al., 2016; Jones et al., 2014; van den Bos et al., 2012; Westhoff et al., 2021). In a social learning task where participants made predictions about the preferences of peers, activation in the fusiform cortex was associated with PEs (Rosenblau et al., 2018). In terms of age-related differences, studies reported 1) no age-related change in PE responses when learning for self (Westhoff et al., 2021), 2) peak striatal activity in adolescence (Cohen et al., 2010), 3) greater hippocampal PE-related activity in adolescents vs. adults (Davidow et al., 2016), and 4) greater activation in insula with positive PEs specific to adolescents (Jones et al., 2014). The expected values and predictions in these tasks correlated with the medial PFC responses, which were stronger in adults relative to adolescents (Jones et al., 2014; Rosenblau et al., 2018). In addition, one study investigating the functional connectivity between striatum and medial PFC reported enhanced connectivity during the receipt of positive versus negative feedback, which also increased with age (van den Bos et al., 2012).

Taken together, these findings are difficult to reconcile in terms of systematic developmental changes. The reported inconsistencies seem due to the variety of tasks (e.g., some requiring higher working-memory capacity or social tasks) and computational models (e.g., single vs. asymmetrical learning rates) used as well as sample characteristics. For example, there are inconsistencies in the cutoff ages that different studies used in order to group participants as children, adolescents, and adults. Combined with differences in analytic approaches (e.g., age used as a continuous variable vs. grouping variable), such sample differences may have contributed to mixed findings when comparing ages. Despite this, most studies using stable probabilistic learning tasks that involve expected uncertainty (due to outcome stochasticity) somewhat consistently reported a decrease in choice stochasticity (Decker et al., 2015; Palminteri et al., 2016; Westhoff et al., 2021; Xia et al., 2021 but see Davidow et al., 2016; and van den Bos et al., 2012) and an increase in performance from childhood to adulthood (Decker et al., 2015; Hämmerer et al., 2011; Jones et al., 2014; Palminteri et al., 2016; Rosenblau et al., 2018; Westhoff et al., 2021; Xia et al., 2021 but see Davidow et al., 2016; Raab & Hartley, 2020; and Smith et al., 2012).

### Results: Development of learning from volatile outcomes

Dynamic and volatile environments contain reversals or sudden changes in the outcome statistics that evoke unexpected uncertainty. In these environments, typically the challenge is to optimally respond to unexpected outcomes, because they might signal either a change or occur due to stochasticity or noise in the environment. Table 2 shows the overview of developmental studies that used tasks with high volatility, such as probabilistic or deterministic reversal learning tasks (*n* = 6) and a predictive inference task (*n* = 1; Bruckner et al., 2020). The majority (*n* = 6) of these studies also employed computational models to analyze the behavioral patterns. All studies recruited adolescents. Except for one study, which compared children to adolescents (Weiss et al., 2021), the others compared younger participants to adults. A subset of studies (*n* = 3) included neuroimaging findings. Except for one experimental condition in one of the reported studies (i.e., videos showing an individual smiling and giving thumbs up; Weiss et al., 2021), all studies with volatile outcomes focused on learning from nonsocial reward or feedback.

Similar to tasks that involved outcome stochasticity but not volatility, these studies using tasks with higher volatility also reported mixed findings regarding age-related differences in the patterns of learning rates. Some studies reported no differences in learning rates (Bruckner et al., 2020; Javadi et al., 2014; Waltmann et al., 2023). Others reported higher learning rates in adolescents compared with adults (particularly for negative outcomes; Hauser et al., 2015), or found higher learning rates in adolescents than in children (Bruckner et al., 2020; Weiss et al., 2021). In contrast, a recent study reported lowest negative learning rates in adolescents among all the age groups (Eckstein et al., 2022). Only two of these studies modeled and reported on both positive and negative learning rates (Eckstein et al., 2022; Hauser et al., 2015), whereas others reported on a single learning rate.

With regard to the inverse temperature parameter, these studies reported either no age-related differences (Hauser et al., 2015; Weiss et al., 2021) or increases with age (Eckstein et al., 2022; Javadi et al., 2014), indicating less exploration or noisy choices with age. A recent study found that adolescents were less sensitive to particularly positive reinforcement than adults, which leads adolescents to show more response switching akin to more exploratory/noisy choice behavior (Waltmann et al., 2023). In addition, whereas some studies reported peak performance in adolescence compared with other ages (Eckstein et al., 2022; van der Schaaf et al., 2011; Weiss et al., 2021), or better performance of adolescents than adults in early trials of more volatile phases (Waltmann et al., 2023), the others did not find any differences in performance between adolescents and adults (Bruckner et al., 2020; Hauser et al., 2015; Javadi et al., 2014).

Neuroimaging findings show that in a learning context with high volatility, PEs were found to be associated with the activity in the striatum, ventral medial PFC, and posterior cingulate cortex, yet with neglectable or very limited age-related differences (Hauser et al., 2015; Javadi et al., 2014; Waltmann et al., 2023). One study reported an increased right insula response to negative PEs in adolescents compared with adults (Hauser et al., 2015). Another study reported that activity in the medial PFC scaled with choice probability predicted by the computational model and was stronger in adults than adolescents (Waltmann et al., 2023).

Although all these studies involved volatility, the characteristics of the experimental paradigms, computational models used, and samples varied considerably. This group of studies most commonly employed probabilistic learning tasks. However, even when we only compare the probabilistic reversal tasks that involve choosing between two options, the exact probabilities associated with a given outcome were 80%, 75%, or 60% in different studies. The outcomes could be gain and loss, gain and no gain, or loss and no-loss. In addition, there were inconsistencies in the cutoffs used to define different age groups along with differences in the analytic approach to assess age-related effects in these studies similar to those in studies that employed tasks with stochastic outcomes. Despite these differences, it seems that adolescents either performed comparable to adults (Bruckner et al., 2020; Hauser et al., 2015; Javadi et al., 2014) or better (Eckstein et al., 2022; van der Schaaf et al., 2011; during early reversal phases in Waltmann et al., 2023; recently similar findings were reported in Chierchia et al., 2022) in such dynamic and changing environments with higher levels of volatility.

### Interim summary: Comparing the development of learning from stochastic to volatile outcomes

The results suggest that adolescents might have an advantage over younger and older age groups when learning in dynamic environments with volatile outcomes. Particularly, they might perform relatively better than adults when learning from volatile outcomes compared with learning from only stochastic outcomes where they generally seem to perform worse than adults. More specifically, among the studies, three of six that included adolescents and adults showed that adolescents were better at learning from volatile outcomes than adults (note that this was only true in early reversal phases in Waltmann et al., 2023); the other three showed no age-related changes from adolescence to adulthood. Interestingly, none of these studies reported that adolescents performed worse (i.e., fewer correct choices) at learning from volatile outcomes. Although the results for stochastic outcomes appear to be somewhat mixed, there seemed to be an improvement in learning from stochastic but stable outcomes with age. Eleven of 17 studies that reported on learning performance showed that adolescent's learning improved with age, whereas two studies found an age-related decline and three did not find age-related differences in performance between adolescents and adults.

Only 10 among the 26 studies reviewed included neuroimaging data. Moreover, the heterogeneity of the learning paradigms and modeling approaches in the reviewed studies makes it difficult to compare the neural correlates of the processes involved in learning under stochastic and volatile outcomes. Interestingly, most of these studies do not find age-related differences in the processing of PEs in these regions (but see Cohen et al., 2010). Also, we did not identify any regions that dissociated learning from stochastic outcomes and volatile outcomes. One explanation is that these learning processes may overlap and depend on the same learning systems in the brain. Alternatively, different levels of volatility (or surprise) may target more specific neural systems, although there are limited indications for a distinction in learning systems in the developmental comparison we included.

## Discussion and future directions

### Mechanisms underlying adolescent learning and decision-making under different types of uncertainty

From this review, our findings indicate that adolescents, compared with children and adults, seem to have a relative advantage when learning from volatile outcomes. When learning from stochastic outcomes adolescents, compared with adults, have a relative disadvantage, but future studies are needed to identify the underlying causal mechanisms for this effect. These empirical studies may suggest at least several candidate mechanisms. First, although the findings for learning rates were largely mixed, explorative or noisy choices—e.g., as indicated by the (inverse) temperature parameter—decreased from adolescence to adulthood across learning tasks (Nussenbaum & Hartley, 2019). On average, these exploratory or noisy choices can result in gaining less reward or incurring greater loss in environments where outcomes are stochastic, but stable. However, in environments where outcomes are volatile, these choices can result in faster detection of changes in outcomes, as the lower value options keep being sampled instead of avoided entirely (Denrell, 2005; Fan et al., 2022; Lloyd et al., 2022). The decrease in exploratory or noisy choices with age is therefore one potential mechanism by which adolescents might perform better when learning from volatile outcomes than when learning from only stochastic outcomes relative to adults.

Second, adolescents may be more prone to perceive volatility in environments in which outcomes are in reality only stochastic (Jepma et al., 2020). This could indicate that adolescents either expect more volatility in their environment or that they mistake stochastic outcomes as signals of volatility. Furthermore, adolescents who estimate higher volatility in such environments may have both higher learning rates and engage in more exploratory or noisy choices (Jepma et al., 2020). The majority of studies we reviewed employed learning tasks with a choice component. In such tasks, both the updating of the expected values and choice behavior play a role in determining performance. Thus, making use of designs that examine these processes partially independently (e.g., an estimation task without a choice function, and a probabilistic learning task with a choice function as in Jepma et al., 2020) under different types of outcome uncertainty may help better understand the mechanisms that give rise to age-related differences in performance when learning from volatile and stochastic outcomes.

Finally, neurobiological, hormonal, and environmental changes that take place during adolescence may explain why the developmental changes in learning from stochastic and volatile outcomes occur and relate to the changes in exploration, noise, and perceptions of volatility. In a developmental perspective, an interesting question for future research is therefore whether, for instance, pubertal onset is tied to these cognitive computations and expectations. In addition, studies have suggested that pubertal changes may initiate a cascade of neurobiological changes that influence learning and brain plasticity, including for instance dopamine functioning (Larsen & Luna, 2018). More research is needed to combine learning in stochastic and volatile environments to these developmental changes in hormonal and neurotransmitter functioning. Longitudinal studies, in particular, could be crucial in differentiating the effects of age versus puberty on learning in diverse uncertain situations.

### Understanding the links between volatility, exploration, and noise in empirical studies

In decision making, noise refers to random fluctuations that can affect the accuracy and consistency of our judgments or actions, whereas exploration refers to the process of seeking out new information or options to improve our understanding of a situation, which would then be used to identify better courses of action. Across our reviewed studies, one of the more consistent developmental differences in computational model parameters was observed in the inverse temperature. Although historically considered to reflect exploration (Daw et al., 2006), this parameter could be interpreted as a form of exploration, as decision noise, and sometimes these accounts are difficult to distinguish.

Recent frameworks, such as those proposed by Gershman (2018) and Wilson et al. (2014), provide a more nuanced view on exploration and are promising for future developmental studies. For example, some decision contexts may call for directed exploration (e.g., when there is relative uncertainty, the more uncertain option may be favored). Other decision contexts call for random exploration (e.g., when the total uncertainty is high, not dependent on relative uncertainty) (Fan et al., 2022; Gershman, 2018; Tomov et al., 2020). When the outcomes of the options are volatile as opposed to only stochastic, this also leads to more exploration (Fan et al., 2022). Random exploration is thought to be stable across development, but interestingly, the strategic use of directed exploration has been suggested to emerge across adolescence (Somerville et al., 2017). This puts forward a promising hypothesis regarding age-related changes in goal-directed exploration and its interactions with outcome uncertainty, which can be targeted by using specific experimental paradigms and models (Fan et al., 2022; Tomov et al., 2020).

Another recent framework that would be interesting to test using a developmental perspective disentangles *decision* noise from *computation* noise (Findling et al., 2019). According to this framework, the variability in choice behavior that would be traditionally attributed to decision noise (or “exploration”) could be, to a large degree, explained by noise in the *updating* of the action values (i.e., computation noise; Findling et al., 2019; Findling & Wyart, 2021). An interesting feature of computation noise is that it increases with the magnitude of the prediction errors, particularly in volatile environments (Findling & Wyart, 2021). The potential benefits of increased computation noise in volatile environments could be to support the flexibility to adapt to unpredictable changes or balancing the cost of computation precision. It is a possibility that the increased choice stochasticity in adolescents that we observed in the studies reviewed also can be attributed to computation noise. However, no study to date has directly examined age-related changes in computation noise. This remains to be addressed in future studies.

One important point to consider is that several of the tasks analyzed in our review, including those that involve volatile task environments (e.g., implementing a mean-level shift in the reward structure), typically incorporate stochastic elements (Fig. 1; Supplementary Table S3). At the moment almost no developmental studies explicitly estimate stochasticity and volatility (but see Jepma et al., 2020). However, such an approach is important when the goal is to understand the mechanisms that give rise to behavioral differences when learning from outcomes that involve different types of uncertainty. For example, adolescents may be more prone to perceive volatility in environments in which outcomes are, in reality, only stochastic (Jepma et al., 2020). Recent models that explicitly estimate both stochasticity and volatility (Piray & Daw, 2021) can be combined with paradigms that manipulate stochasticity and volatility within the same individuals while keeping other task characteristics as similar as possible (Behrens et al., 2007). These experimental and methodological advances would allow us to examine more directly the developmental differences in learning under stochasticity and volatility.

Testing these frameworks also requires studies with larger samples or multiple studies using the same task and models. As mentioned in our interim summary and Table 1 (see also Supplementary Table S3 for reported means of parameter values), most studies include different paradigms with slightly different computational approaches, making it difficult to directly compare parameter values across studies. The issue of generalizability of computational approaches is clearly outlined in previous reviews (Eckstein et al., 2021; 2020). In brief, their findings show that in many cases, computational parameters cannot be directly compared between studies, because the processes that are captured depend on task characteristics, such as feedback valence, memory load, choice of parameters, volatility, and others. We therefore capitalized on a comparison of age (groups) within studies and subsequently summarized these findings for task environments that differ in volatility versus stochasticity. Also, differences between studies may occur due to variations in sample characteristics. Studies covering the period of adolescence have relied on different age ranges and cutoffs (Table 1), in which the youngest adolescents included were aged 10, 12, 13, or 14 years and the oldest were aged 14, 15, 17, 18, and 19 years. Finally, although not reported here, socioeconomic status, or education levels may differ between studies and/or age groups. For future studies, these sample characteristics are important to consistently report in the literature (Qu et al., 2021).

### Individual differences in learning and decision-making with uncertainty

The developmental studies that were identified in this review largely ignored the subjective experience of uncertainty. Uncertainty is perceived to be threatening by most people and is associated with stress (de Berker et al., 2016; Grupe & Nitschke, 2013; Peters et al., 2017). Anxious individuals have been shown to have difficulties processing the cues in their environment to estimate the type of uncertainty and adjust their learning accordingly (Piray & Daw, 2021; Pulcu & Browning, 2019). For example, individuals with higher trait anxiety and transdiagnostic anxious and depressive symptomatology showed little difference in learning rates between volatile compared with stable (stochastic) environments, whereas optimal learners increase their learning rates (i.e., learn faster) in volatile environments (Browning, 2015; Gagne et al., 2020). According to a recent conceptual framework (Piray & Daw, 2021), learners simultaneously make inferences about the stochasticity and volatility in an environment, which are compensatory processes influencing the adjustment of learning rates. Within this framework, anxiety is suggested to be mainly associated with the maladaptive functioning of the processes involved in stochasticity inference such that anxious individuals assume higher volatility in environments that are actually stable but highly stochastic. Alternatively, anxiety might be related to reduced exploration and adaptation of exploratory behavior to volatility where exploring more might be beneficial (Fan et al., 2022; Lloyd et al., 2022). Anxiety and depressive symptoms are particularly relevant to include from a developmental perspective, as the onset of anxiety disorders and depression are most prevalent during adolescence (Blakemore, 2019; de Lijster et al., 2017; Kessler & Bromet, 2013; McLaughlin & King, 2015). To what extent uncertainty, and the mechanisms that may drive the experience of uncertainty, play a role in the development of mental health symptomatology is an important question for future developmental studies. A longitudinal perspective will be crucial to unravel who is at risk for developing mental health illnesses.

### Uncertainty in social environments

Our review includes paradigms that examine learning in both social and nonsocial environments. However, most studies in our review use abstract paradigms, which necessitate individuals to learn a stimulus-outcome association based solely on their personal experiences without any social cues. Although learning through prediction errors can occur in both social and nonsocial contexts (Ruff & Fehr, 2014), learning in a social context may sometimes involve different strategies than learning in a nonsocial context (Hackel et al., 2020). Also, many of the uncertainties that adolescents learn to navigate in this phase of life stem from social interactions as they begin to interact with their environments as autonomous individuals, take on various social roles, and form new friendships and romantic relationships (Crockett & Crouter, 2014). Moreover, adolescence is a developmental phase in which social reorientation takes place such that importance of peers and salience of social information becomes more prominent (Crone & Dahl, 2012; Nelson et al., 2005, 2016). This supports the relevance of learning and decision-making in social contexts given that adolescents’ understanding of what their peers value and think should considerably weigh into their value estimations and influence their decisions (Pfeifer & Berkman, 2018). It is therefore important for adolescents to learn about and update their knowledge of the characteristics of other people (e.g., what and whom they like; Jones et al., 2014; Rosenblau et al., 2018, or how trustworthy they are; Ma et al., 2022b) and social groups (e.g., how cooperative or trustworthy they are; Westhoff et al., 2020). For building social ties, it is important to learn how consequences of our actions influence ourselves and others (e.g., whether our actions are harmful for others; Westhoff et al., 2021) or by observing others and benefiting from their experiences (Rodriguez Buritica et al., 2019). There have been efforts to address the importance of studying the uncertainty processing in social contexts during adolescence (Blankenstein et al., 2016; Hofmans & van den Bos, 2022; Ma et al., 2022b). For example, one study used computational models to examine uncertainty in social contexts directly and found that adolescents have weaker prior expectations about the social behavior of their peers, which resulted in faster learning about their peers (Ma et al., 2022b).

Additionally, it has been suggested that adolescents’ ability to adapt to volatile social environments may manifest in the increased variability of their moods (Gregorova et al., 2022). For example, their positive or negative mood may signal a general increase or decrease of social rewards in the adolescent environment, thereby facilitating quick adjustment to interactions with friendly (positive mood) or hostile (negative mood) others. However, in cases where one’s mood largely biases their learning or where one engages in suboptimal learning (e.g., estimating higher volatility in an environment with stable but stochastic outcomes), increased mood variability may pose a risk for mental health problems (Gregorova et al., 2022). Taken together, future research is needed to unpack how the uncertainty in adolescents’ social environments may provide rich and adaptive opportunities for learning.

## Conclusions

The ability to tailor learning and decision-making under uncertainty is crucial for adaptive behavior, especially given that uncertainty is intrinsic to most real-life situations. In this review, we discussed different types of uncertainty, focusing mainly on two types of outcome uncertainty: stochasticity and volatility, as these have different influences on learning and decision-making. Taking a developmental approach, with a focus on adolescence as a period characterized by change and uncertainty, we summarized the recent findings from studies that compared different age groups in learning tasks that involved different types of uncertainty. While we observed that the findings were mixed, there were interesting consistencies in the age-related differences in model parameters and performance. The findings suggest that the development of learning under uncertainty might depend on the statistics of the environment and the type of uncertainty that the individual is exposed to. Interestingly, adolescents may have an advantage when learning from volatile outcomes. In contrast, adolescents’ more exploratory or noisy choice behavior seems a disadvantage when learning from stochastic outcomes in relatively stable contexts. This is possibly an adaptive response to the rather complex and continuously changing environments that adolescents encounter in real life. Future studies are needed to test this relationship more directly and expose mechanisms through which adolescents gain this advantage in learning. Together, these findings contribute to the understanding of adolescence as a sensitive period for learning in uncertain and dynamically changing environments.

## Supplementary Information

ESM 1 (DOCX 207 kb)
