# Supplementary material for: Expecting the unexpected: a review of learning under uncertainty across development
Source: Cogn Affect Behav Neurosci. 2023 May 26;23(3):718–38. doi: 10.3758/s13415-023-01098-0 (PMC10390612; doi:10.3758/s13415-023-01098-0)
Supplement: Supplementary file 1 — (DOCX 207 kb) [file 13415_2023_1098_MOESM1_ESM.docx]

**Supplementary Materials**

**Table S1**. Table showing search terms for each aspect we combined

| Aspect 1: | "Uncertainty"[Mesh] OR "Probability Learning"[Mesh] OR "Reversal Learning"[Mesh] OR "Exploratory Behavior"[Mesh] OR "Risk-Taking"[Majr:NoExp] OR "Reinforcement, Psychology"[Mesh:NoExp] OR "Probabilistic Learning"[tiab] OR "Probability Learning"[tiab] OR "Reinforcement Learning"[tiab] OR "Explore-Exploit"[tiab] OR "Explore Exploit"[tiab] OR "Learning Rate*"[tiab] OR "Inverse Temperature"[tiab] OR "Choice Stochasticity"[tiab] OR "Random Exploration"[tiab] OR "Directed Exploration"[tiab] |
| --- | --- |
| Aspect 2: | "Psychology, Developmental"[Mesh] OR "Adolescent"[Mesh] OR "Adolescent Development"[Mesh] OR "Young Adult"[Mesh] OR "Puberty"[Mesh] OR "Brain/growth and development"[Mesh] OR "Adolescen*"[tiab] OR "pubert*"[tiab] OR "juvenile*"[tiab] OR "teen"[tiab] OR "teens"[tiab] OR "teenager*"[tiab] OR "youth*"[tiab] or "young people"[tiab] OR "young adult*"[tiab] OR "pubescent*"[tiab] OR "young women"[tiab] OR "young men"[tiab] |
| Aspect 3: | "Magnetic Resonance Imaging"[Mesh] OR "Models, Neurological"[Mesh] OR "Models, Psychological"[Mesh:NoExp] OR "Pupil/physiology"[Mesh] OR "Bayes Theorem"[Mesh] OR "Brain"[Mesh] OR "fMRI"[tiab] OR "neural"[tiab] OR "brain imag*"[tiab] OR "magnetic resonance imag*"[tiab] OR "Neurobiolog*"[tiab] OR "brain"[tiab] OR "neuroimag*"[tiab] |
| Aspect 4: | "Anxiety"[Mesh] OR "Anxiety Disorders"[Mesh] OR "Depression"[Mesh] OR "Depressive Disorder"[Mesh] OR "Impulsive Behavior"[Mesh] OR "Internalizing*"[tiab] OR "Externalizing*"[tiab] OR "Depressi*"[tiab] OR "Anxi*"[tiab] OR "Impulsiv*"[tiab] |

**Table S2.** Table showing the order of the search queries and number of hits

| Search | Query | Items found |
| --- | --- | --- |
| #1 | your Aspect 1 NOT ("Animals"[Mesh] NOT "Humans"[Mesh]) | 48,948 |
| #2 | your Aspect 2 NOT ("Animals"[Mesh] NOT "Humans"[Mesh]) | 2,860,844 |
| #3 | your Aspect 3 NOT ("Animals"[Mesh] NOT "Humans"[Mesh]) | 1,752,918 |
| #4 | Combine #1 and #2 | 14,206 |
| #5 | #4 Filters: from 2010 - 2022 | 9,064 |
| #6 | Combine #1 and #2 and #3 Filters: from 2010 – 2022 NOT (Review[Publication Type]) | 1,863 |

**
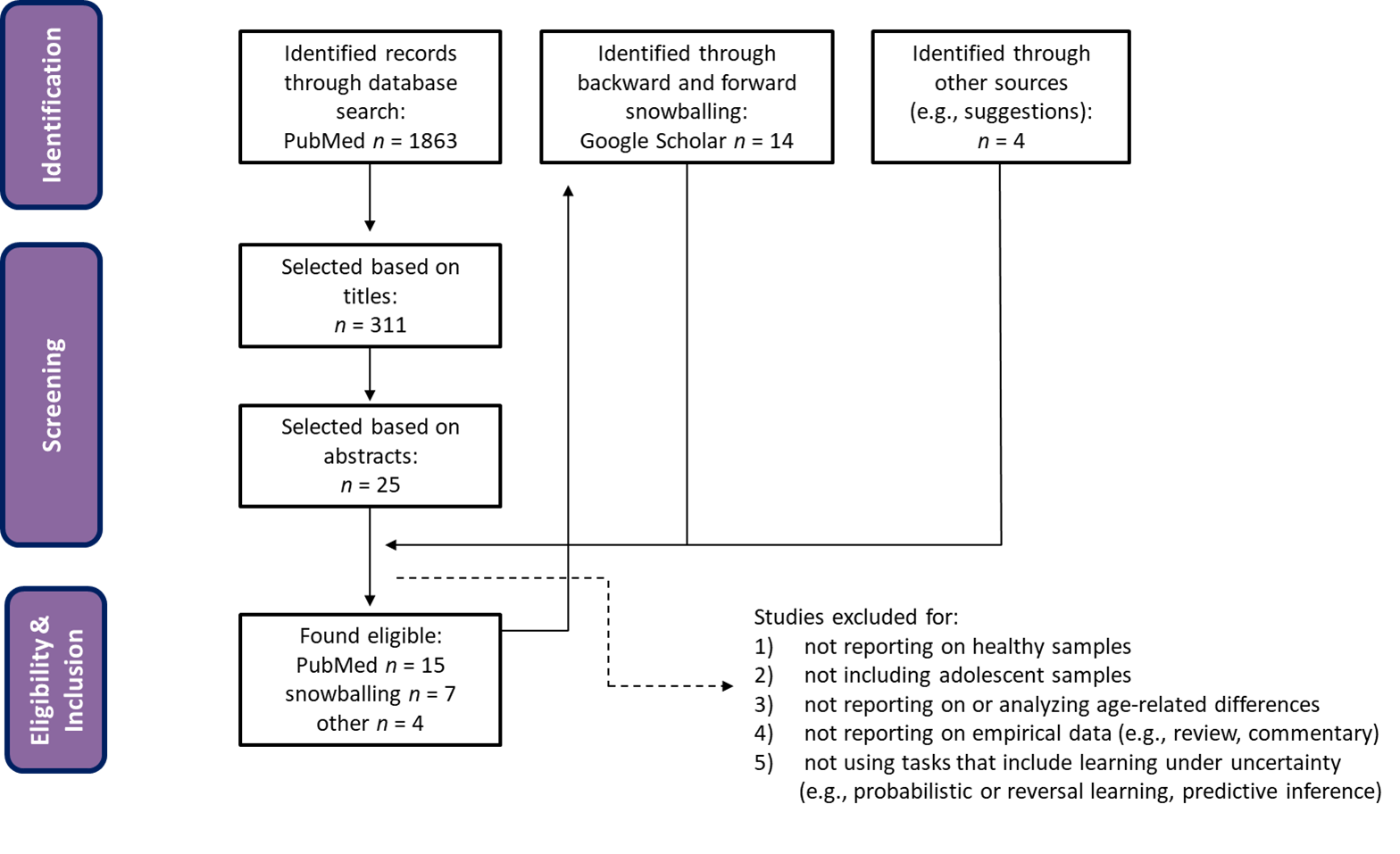
**

**Figure S1.** Flow diagram showing the process of identifying and including studies to review. Following the first step of identifying eligible studies through a search in a scholarly database; these identified studies were used as seed references for forward and backward snowballing.

**Table S3.** Table showing the descriptives (mean or median) of parameters reported in the studies reviewed.

| Authors (Year) | Task Type | Age Group (N) | Mean Parameter Values* | Outcome Uncertainty Type |
| --- | --- | --- | --- | --- |
| Christakou et al. (2013) | Adapted Iowa Gambling Task (IGT) | 11.9 – 31.2 (N_adolescents_ = 18;  N_adults_ = 19) | Learning rates:  Not reported  Inverse temperature:  adolescents: ~1  adults: ~1.5 | Stochasticity |
| Cohen et al. (2010) | Probabilistic learning | children: 8-12 (N = 18);  adolescents: 14-19 (N = 16);  adults: 25-30 (N = 11) | Learning rates:  children: 0.55;  adolescents: 0.55;  adults: 0.51  Inverse temperature:  NA | Stochasticity |
| Davidow et al. (2016) | Probabilistic learning | adolescents : 13-17 (N = 41 ; N_fMRI_ = 25) ;  adults : 20-30 (N = 31 ; N_fMRI_ = 22) | Learning rate:  adolescents: 0.38;  adults: 0.55  Inverse temperature:  adolescents: 5.6;  adults: 6.1 | Stochasticity |
| Decker et al. (2015) | Instructed probabilistic learning | children: 6-12 (N = 30); adolescents: 13-17 (N = 31); adults: 18-34 (N = 26) | Standard RL:  Learning rate:  children: 0.43;  adolescents: 0.08;  adults: 0.04  Inverse temperature:  children: 0.80;  adolescents: 1.25;  adults: 3.89  Modified bias RL  Learning rate:  children: 0.29;  adolescents: 0.05;  adults: 0.05  Inverse temperature:  children: 1.32;  adolescents: 2.89;  adults: 4.55 | Stochasticity |
| Hämmerer et al. (2011) | Probabilistic learning | children: 9-11 (N = 44); adolescents: 13-14 (N = 45); younger adults: 20-30 (N = 46); older adults: 65-75 (N = 44) | NA | Stochasticity |
| Humphreys et al. (2016) | Balloon Emotional Learning Task (BELT) (Modified Balloon Analogue Risk Task [BART]) | children & adolescents: 3-17 (N = 140);  adults: 18-36 (N = 76) | NA | Stochasticity |
| Jepma et al. (2020) | Estimation and choice tasks | adolescents: 12-15 (N = 25);  adults: 18-29 (N = 35) | Learning rate:  Not reported  (for the same model)  Assumed volatility  (per noise condition):  adolescents: 0.02 & 0.001 ;  adults : 0 & 0  Inverse temperature:  (dynamic – consists of two parameters)  Change parameter:  adolescents: 0.74;  adults: 0.79  Inverse temperature on trial 10:  adolescents: 0.14;  adults: 0.22 | Stochasticity |
| Jepma et al. (2022) | Experience-based risk taking | early adolescents : 12-14 (N = 31);  mid-late adolescents : 15-17 (N = 39);  adults : 20-35 (N = 35) | Learning rate:  Early adolescents: ~0.6;  Mid-late adolescents: ~0.3;  Adults: ~0.1  Inverse temperature:  Early adolescents: ~0.1;  Mid-late adolescents: ~0.2;  Adults: ~0.8 | Stochasticity |
| Jones et al. (2014) | Social evaluative  reinforcement learning | 8-25 (N = 120; N_fMRI_ = 87) | Positive learning rate:  Not reported  Negative learning rate:  Not reported  Inverse temperature:  NA | Stochasticity |
| Nussenbaum et al. (2022) | Adapted IGT | children: 8-12 (N = 47);  adolescents: 13-17 (N = 46);  adults: 18-25 (N = 49) | Positive learning rate:  children: ~0.3;  adolescents: ~0.3;  adults: ~0.25  Negative learning rate:  children: ~0.2;  adolescents:~0.2;  adults:~0.1  Inverse temperature:  Not reported | Stochasticity |
| Palminteri et al. (2016) | Instrumental probabilistic RL | adolescents: 12-17 (N = 26)  adults: 18-32 (N = 24) | Learning rate:  adolescents: 0.38;  adults: 0.39;  Inverse temperature:  adolescents: 2.73;  adults: 3.90 | Stochasticity |
| Raab & Hartley (2020) | Probabilistic Go/No-Go learning | children : 8 – 12 (N = 20) ; adolescents : 13 – 17 (N =20);  adults : 18 – 25 (N = 21) | Learning rate:  (median)  children: 0.22;  adolescents: 0.36;  adults: 0.48;  Reinforcement sensitivity:  (median)  children: 3.16;  adolescents: 4.85;  adults: 4.51;  Inverse temperature :  NA | Stochasticity |
| Rodriguez Buritica et al. (2019) | Adapted IGT | children: 8-10 (N = 24);  adolescents: 13-15 (N = 24);  adults: 18-22 (N = 25) | Positive learning rate:  children: ~0.3;  adolescents: ~0.15;  adults: ~0.3;  Negative learning rate:  children: ~0.3;  adolescents: ~0.1;  adults: ~0.1;  Inverse temperature:  children: ~0;  adolescents: ~0;  adults: ~0.1; | Stochasticity |
| Rosenblau et al. (2018) | Social learning /  Theory of Mind (ToM) | adolescents : 10-17 (N =24) ;  adults : 23-36 (N = 21) | Learning rate :  adolescents: ~0.15;  adults: ~0.3  Inverse temperature:  NA | Stochasticity |
| Smith et al. (2012) | computerized IGT | 8-17 (N = 122) | NA | Stochasticity |
| van den Bos et al. (2012) | Probabilistic learning | children: 8-11 (N = 18); adolescents: 13-16 (N = 27); adults: 18-22 (N = 22) | Positive learning rate:  children: ~0.25;  adolescents: ~0.25;  adults: ~0.38  Negative learning rate:  children: ~0.4;  adolescents: ~0.25;  adults: ~0.15  Inverse temperature:  Not reported | Stochasticity |
| Westhoff et al. (2020) | Repeated probabilistic  trust learning | 8-23 (N = 244) | Learning rate:  (consists of a start LR and decay parameter [τ]).  Trust game  ages 8-11: LR: 0.12, τ: 0.00  ages 12-14: LR: 0.82, τ: 1.27  ages 15-18: LR: 1.00, τ: 5.00  ages 19-23: LR: 0.96, τ: 5.00  Coordination Game  ages 8-11: LR: 0.67, τ: 1.94  ages 12-14: LR: 0.61, τ: 1.31  ages 15-18: LR: 1.00, τ: 5.00  ages 19-23: LR: 0.96, τ: 5.00  Non-social learning task  ages 8-11: LR: 0.14, τ: 2.99  ages 12-14: LR: 0.06, τ: 1.80  ages 15-18: LR: 0.05, τ: 1.87  ages 19-23: LR: 0.20, τ: 1.43)  Inverse temperature:  Not reported | Stochasticity |
| Westhoff et al. (2021) | Prosocial learning | 9-21 (N = 74) | Learning rate:  Self:  Ages 9-11: ~0.35  Ages 13-17: ~0.3  Ages 19-21: ~0.2  Other:  Ages 9-11: ~0.4  Ages 13-17: ~0.3  Ages 19-21: ~0.25  No one:  Ages 9-11: ~0.3  Ages 13-17: ~0.35  Ages 19-21: ~0.3  Inverse temperature:  Self:  Ages 9-11: ~6  Ages 13-17: ~9  Ages 19-21: ~11  Other:  Ages 9-11: ~4  Ages 13-17: ~8  Ages 19-21: ~10  No one:  Ages 9-11: ~6  Ages 13-17: ~7  Ages 19-21: ~9 | Stochasticity |
| Xia et al. (2021) | Probabilistic learning | children & adolescents: 8-17 (N = 157);  adults: 18-30 (N = 118) | Positive learning rate:  overall: 0.18;  Inverse temperature:  overall: ~11 | Stochasticity |
| Bruckner et al. (2020) | Predictive inference | children: 8-10 (N = 33);  adolescents : 12-17 (N = 29);  young adults: 20-28 (N = 32);  older adults: 62-80 (N = 35); 2^nd^ study:  children: 7-11 (N = 31)  young adults: 20-28 (N = 25)  older adults: 61-76 (N = 34) | Learning rate:  Children: 0.73;  older adults: 0.79;  adolescents: 0.85;  younger adults: 0.84  Inverse temperature:  NA | Volatility (in addition to stochasticity) |
| Eckstein et al. (2022) | Probabilistic (reward)  reversal learning | children & adolescents: 8-18 (N = 179);  adults: 18-30 (N = 112) | Positive learning rate:  overall: ~ 0.73  Negative learning rate:  overall: ~ 0.60  Inverse temperature:  overall: ~ 3.48 | Volatility (in addition to stochasticity) |
| Hauser et al. (2015) | Probabilistic reversal learning | adolescents: 12-16 (N = 19);  adults: 20-19 (N =17) | Positive learning rate:  adolescents: 0.69;  adults: 0.49  Negative learning rate: adolescents: 0.62;  adults: 0.45;  Inverse temperature:  adults: 2.4;  adolescents: 1.9 | Volatility (in addition to stochasticity) |
| Javadi et al. (2014) | Probabilistic reversal learning | adolescents: 14-15 (N = 219);  adults: 20-39 (N = 29) | Learning rate:  adolescents: 0.31;  adults: 0.27;  Inverse temperature:  adolescents: 0.14;  adults : 0.33 | Volatility (in addition to stochasticity) |
| van der Schaaf et al. (2011) | Deterministic reversal learning | children: 10-11 (N = 13);  younger adolescents: 13-14 (N = 14);  older adolescents: 16-17 (N = 15);  adults: 20-25 (N = 16) | NA | Volatility |
| Waltmann et al. (2023) | Probabilistic reversal learning | adolescents: (N = 40);  adults: (N = 55) | Learning rate:  overall: ~0.45  Positive reinforcement sensitivity:  overall: ~2  Negative reinforcement sensitivity:  overall: ~0.9  Inverse temperature:  NA | Volatility (in addition to stochasticity) |
| Weiss et al. (2021) | Probabilistic reversal learning | children: 8-12 (N =28) ;  adolescents: 13-17 (N = 25) | Learning rate:  Across all conditions:  children : 0.21;  adolescents: 0.26  Control feedback:  children: 0.18;  adolescents: 0.28  Social feedback:  children: 0.24;  adolescents: 0.25  Individualized feedback: children: 0.21;  adolescents: 0.26  Inverse temperature:  children: 5.96;  adolescents: 5.91 | Volatility (in addition to stochasticity) |

***Note.*** *Where parameter values are entered as non-applicable (NA), either the studies did not employ computational models or the fitted models did not include these parameters. Where the mean values were not explicitly reported in the papers, the parameter values were entered as “not reported” in the table. Parameter values reported approximately (~) are usually gathered from the bar graphs and scatter plots used in the studies to visualize the data.
